# Effect of Duration of LED Lighting on Growth, Photosynthesis and Respiration in Lettuce

**DOI:** 10.3390/plants12030442

**Published:** 2023-01-18

**Authors:** Lyubov Yudina, Ekaterina Sukhova, Ekaterina Gromova, Maxim Mudrilov, Yuriy Zolin, Alyona Popova, Vladimir Nerush, Anna Pecherina, Andrey A. Grishin, Artem A. Dorokhov, Vladimir Sukhov

**Affiliations:** 1 Department of Biophysics, N.I. Lobachevsky State University of Nizhny Novgorod, 603950 Nizhny Novgorod, Russia; 2 Federal State Budgetary Scientific Institution “Federal Scientific Agroengineering Center VIM” (FSAC VIM), 109428 Moscow, Russia

**Keywords:** plant biomass, plant metabolism, plant light absorption, photoperiod, production simulation

## Abstract

Parameters of illumination including the spectra, intensity, and photoperiod play an important role in the cultivation of plants under greenhouse conditions, especially for vegetables such as lettuce. We previously showed that illumination by a combination of red, blue, and white LEDs with a high red light intensity, was optimal for lettuce cultivation; however, the effect of the photoperiod on lettuce cultivation was not investigated. In the current work, we investigated the influence of photoperiod on production (total biomass and dry weight) and parameters of photosynthesis, respiration rate, and relative chlorophyll content in lettuce plants. A 16 h (light):8 h (dark) illumination regime was used as the control. In this work, we investigated the effect of photoperiod on total biomass and dry weight production in lettuce plants as well as on photosynthesis, respiration rate and chlorophyll content. A lighting regime 16:8 h (light:dark) was used as control. A shorter photoperiod (8 h) decreased total biomass and dry weight in lettuce, and this effect was related to the suppression of the linear electron flow caused by the decreasing content of chlorophylls and, therefore, light absorption. A longer photoperiod (24 h) increased the total biomass and dry weight, nevertheless an increase in photosynthetic processes, light absorption by leaves and chlorophyll content was not recorded, nor were differences in respiration rate, thus indicating that changes in photosynthesis and respiration are not necessary conditions for stimulating plant production. A simple model to predict plant production was also developed to address the question of whether increasing the duration of illumination stimulates plant production without inducing changes in photosynthesis and respiration. Our results indicate that increasing the duration of illumination can stimulate dry weight accumulation and that this effect can also be induced using the equal total light integrals for day (i.e., this stimulation can be also caused by increasing the light period while decreasing light intensity). Increasing the duration of illumination is therefore an effective approach to stimulating lettuce production under artificial lighting.

## 1. Introduction

Light is a key factor that strongly influences physiological processes in plants, including photosynthesis [1,2,3], which is basis of plant production. It is known that photosynthesis requires light as an energy source [4,5,6,7], can be regulated by light parameters [8,9,10,11,12,13], and is disrupted under the high intensity of light [14,15,16,17]. This means that precision lighting of plants can be effective in improving plant production in cultivated plants [2,3,18]. This method can be based on the plant illumination by LEDs, which generate light with narrow spectral bands and with regulated intensities and time regimes [2,3,18].

Influencing lighting regimes on photosynthetic processes and production in plants can be caused by the direct influence of intensity and spectrum of light on the activity of photosynthetic light reactions and their regulatory processes. It is known that blue and red light are well consumed by photosynthetic light harvesting complexes; however, new works show that green light can also drive light reactions of photosynthesis in leaves [2,3]; therefore, both light spectrum and light intensity can influence the quantity of light absorbed by the photosynthetic machinery. Light-induced changes in photosynthetic processes includes direct changes in photosynthesis and production [5], the activation of regulatory processes (the cyclic electron flow around photosystem I [8,18], energy-dependent components of the non-photochemical quenching of chlorophyll fluorescence in photosystem II [1,10,19,20], “state transition” of the light harvesting complexes [20,21]), and the stimulation of photodamage in the photosynthetic machinery [10,14,18,21].

The complex ways light parameters affect photosynthesis and plant production stress the importance of lighting optimization for cultivated plants, especially for those that are cultivated under artificial lighting, such as lettuce (*Lactuca sativa* L.) [22,23,24,25].

Physiological processes, growth, and production of lettuce plants are strongly affected by illumination spectra: increased intensity of the blue light decreases the biomass, dry weight (DW), light use efficiency, and linear electron flow (LEF), and increases the stomata conductance, cyclic electron flow, dark respiration, and content of chlorophylls and carotenoids [22,23,24,25] whereas the increased intensity of the red light induces the opposite effect.

Light intensity is another important factor for lettuce cultivation; it is known that a higher light intensity increases the biomass, dry weight, total leaf area, photosynthetic CO_2_ assimilation rate (A_hv_), quantum yield of photosystem II (Φ_PSII_), the content of carotenoids, and stomata conductance, and decreases the content of chlorophylls and the non-photochemical quenching of fluorescence [26,27,28]. It should also be noted that excess light is dangerous for plants and induces the photodamage of photosynthetic machinery and the suppression of photosynthesis [1,10]. Lettuce is known to have maximal A_hv_ under the 200 µmol m^−2^s^−1^ light intensity (17 h photoperiod) [28], under the 250 µmol m^−2^s^−1^ light intensity (14 h photoperiod) [28], or under the 350–600 µmol m^−2^s^−1^ light intensity (12 h photoperiod) [26]. Saturation of light dependence of DW of lettuce leaves can be observed under the 200–250 µmol m^−2^s^−1^ light intensity [28].

The influence of photoperiod on physiological, biochemical, and morphological parameters of lettuce seems to be intricate. According to Smirnov et al. [29], an increased photoperiod weakly increases the biomass, DW, and leaf area, stimulates the non-photochemical quenching, suppresses LEF, and decreases the content of chlorophylls. In contrast, studies [28,30] show that the increased photoperiod significantly increases the biomass, DW, content of chlorophylls, stomata conductance, and photosynthetic CO_2_ assimilation. It is not probable that these differences are caused by differences in light spectra because influences of photoperiod under illumination by white fluorescent lamps and under illumination by combination of red and blue LEDs on the chlorophyll content and photosynthetic parameters are similar [30]. In contrast, the light intensity can potentially influence on effect of the photoperiod because the influence of duration of illumination on the chlorophyll content and photosynthesis is weaker in lettuce plants cultivated under 300 μmol m^−2^s^−1^ light and stronger in those cultivated under 150 μmol m^−2^s^−1^ light [28,31]. However, decreasing LEF following increasing photoperiodic conditions was also observed in lettuce plants cultivated under 180 μmol m^−2^s^−1^ light [29].

We previously showed that the artificial illumination based on blue, red, and white LEDs, with high intensity of the red light, could be effective for the lettuce cultivation at the 180 µmol m^−2^s^−1^ total light intensity and 16 h (light):8 h (dark) time regime of illumination [22]. In this work the effect of different photoperiodical conditions on photosynthesis, respiration, biomass and DW of lettuce plants cultivated under the same light spectra were investigated.

## 2. Results

### 2.1. Biometric Determinations

**Figure 1:**
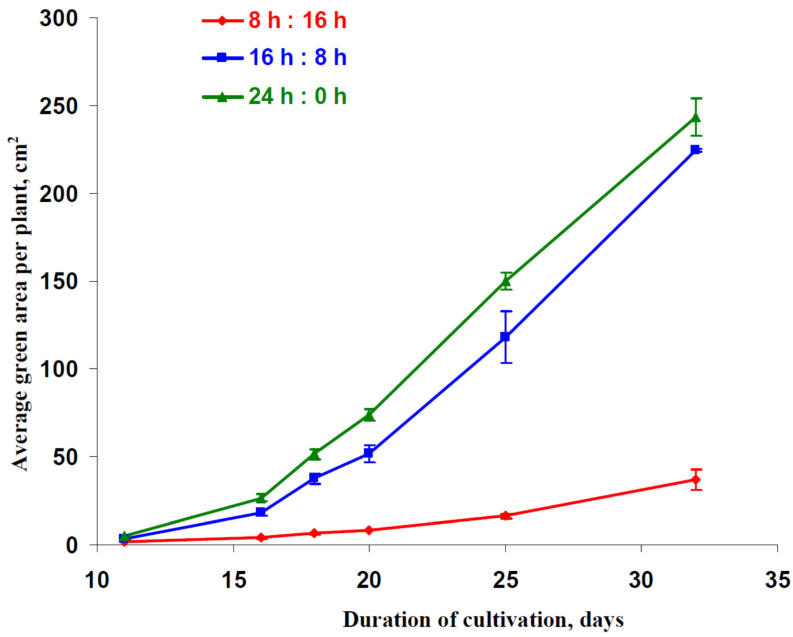
Time dependences of average green area per plant for lettuce cultivated under different photoperiods. “8 h:16 h” is 8 h (light):16 h (dark), “16 h:8 h” is 16 h (light):8 h (dark), and “24 h:0 h” is 24 h (light):0 h (dark). The green area was measured on the basis of a photo of lettuce plants.

The dynamics of an average green area per plant, which were calculated as the ratio of the area of green points in the photo of the pallet with lettuce plants to the number of plants in this pallet, were analyzed on the first stage of the current investigation. This area was related to the total leaf area in the plants which could be illuminated by the direct light from LEDs. Figure 1 reports that increasing photoperiod significantly stimulated the increase of the green area per plant showing an increase in the area of leaves; in contrast, decreasing the photoperiod strongly suppressed this increase.

Table 1 shows the effect of photoperiod on the lettuce production. The 16 h (light):8 h (dark) time regime of illumination was assumed as the control regime in accordance with Yudina et al. [22]. Significant decreases of biomass and DW were observed after 18, 25, and 32 days of the lettuce cultivation under the 8 h photoperiod. In contrast, DW was significantly higher after 18, 25, and 32 days of cultivation under the 24 h photoperiod, even if the biomass increase was not significant.

The relative changes in DW were wider than relative changes in photoperiod. Assuming a total light integral under 16 h photoperiod as the control, integrals under 8 h and 24 h photoperiods were 50% and 150% from the control, respectively. In contrast, relative DWs under 8 h and 24 h photoperiods were 6–12% and 150–430% from the control, respectively. This showed that changes in DW were not proportional to changes in the total light integral.

### 2.2. Photosynthetic Parameters, Relative Content of Chlorophylls and Respiration

The shown dependence of the average green area per plant, biomass, and DW on photoperiod could be caused by changes in photosynthetic processes induced by changes in the illumination duration. Considering this supposition, we investigated the influence of photoperiod on the photosynthetic parameters of leaves. A_hv_ and LEF were investigated because these photosynthetic parameters were strongly related to the activity of photosynthetic dark reactions and, therefore, should influence the production of lettuce plants under their cultivation. A_hv_ and LEF in plants cultivated under the 16 h (light):8 h (dark) time regime of illumination were assumed as the control.

**Figure 2:**
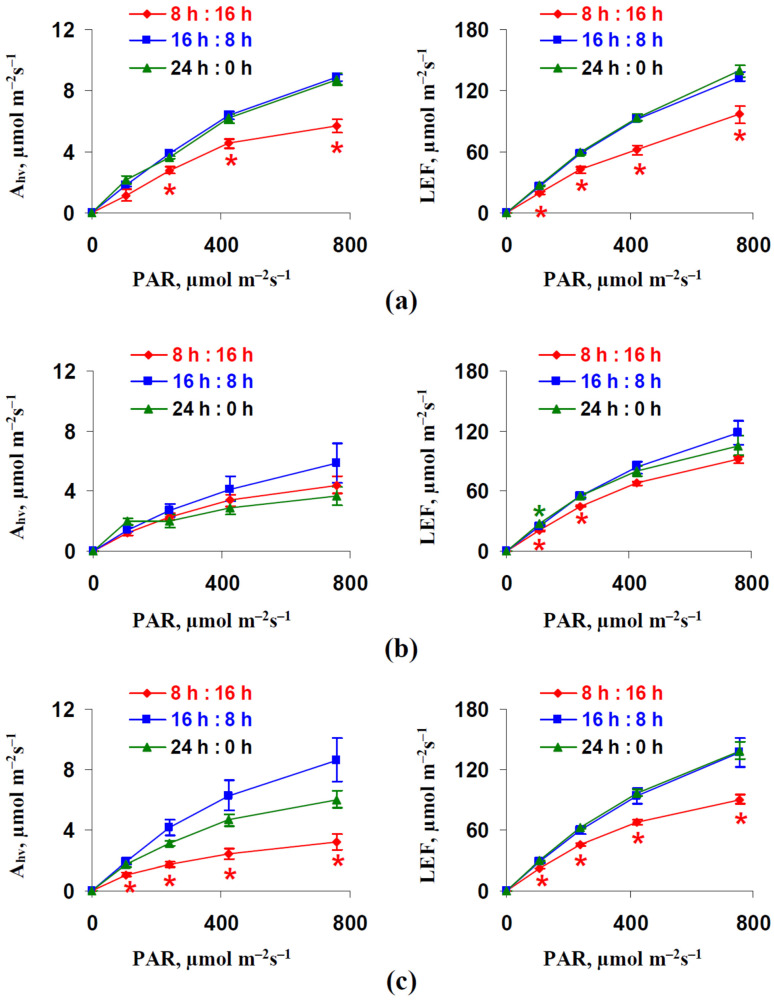
Dependences of the photosynthetic CO_2_ assimilation rate (A_hv_) and linear electron flow (LEF) on intensity of the actinic light (photosynthetically active radiation, PAR) after 18 days (**a**), 25 days (**b**), and 32 days (**c**) of cultivation (*n* = 6). “8 h:16 h” is 8 h (light):16 h (dark), “16 h:8 h” is 16 h (light):8 h (dark), and “24 h:0 h” is 24 h (light):0 h (dark). *, difference from control plants cultivated under 16 h (light):8 h (dark) were significant (*p* < 0.05).

Figure 2 reports that the dependence of the photosynthetic CO_2_ assimilation rate and linear electron flow on intensity of the actinic light (photosynthetically active radiation, PAR) were strongly decreased after 18 and 32 days of the lettuce cultivation under the 8 h photoperiod; these decreases were weak and not significant after 25 days of cultivation. However, increasing A_hv_ and LEF were also absent in plants cultivated under the 24 h photoperiod; moreover, there was a tendency towards decreasing A_hv_ after 25 and 32 days of cultivation in comparison to the photosynthetic CO_2_ assimilation rate in plants cultivated under the 16 h photoperiod. It is known that high intensities of light can induce the photoinhibition of photosynthetic machinery and the disruption of photosynthetic processes [1,10]. As a result, we could not fully exclude the different light sensitivity of photosynthetic machinery in plants cultivated under different photoperiods. However, decreased A_hv_ and LEF were also observed under weak and moderate light intensities (239 and, even, 108 µmol m^−2^s^−1^) which were similar to the intensity of illuminations used for lettuce cultivation. This result showed that photoinhibition did not seem to be the probable mechanism of differences in photosynthetic parameters in plants cultivated under different photoperiods 

**Figure 3:**
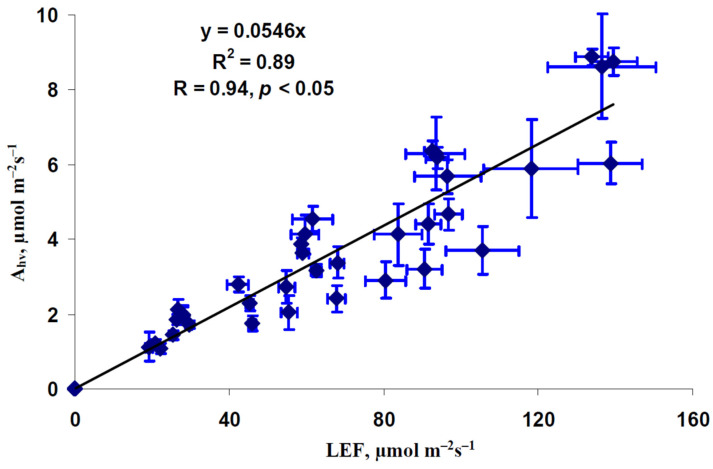
Scatter plot of the photosynthetic CO_2_ assimilation rate (A_hv_) and linear electron flow (LEF) in lettuce plants (*n* = 45). All average values of A_hv_ and LEF from Figure 2 were used. R^2^ and R are the determination and correlation coefficients.

It was believed that changes in A_hv_ could be caused by changes in LEF. Figure 3 shows a scatter plot between the average photosynthetic CO_2_ assimilation rate and linear electron flow. The dependence of A_hv_ on LEF was well described by the linear regression (R^2^ = 0.89). This result was in a good accordance with similar changes in LEF and A_hb_ in work by Yamori et al. [32] and with linear relation between these values showing in literature [33,34] and in our previous work [35]. These results showed that changes in A_hv_ induced by changes in photoperiod could be caused by changes in LEF (at least partially).

It should be noted that Figure 2c supported a rather weak linear relation between A_hv_ and LEF in plants cultivated under the 16 and 24 h photoperiods; this question required additional checking. Figure S1 shows that the correlation coefficient between A_hv_ and LEF (under the 758 µmol m^−2^s^−1^ light intensity) decreased from 0.84 to 0.82 after excluding plants cultivated under the 8 h photoperiod from analysis; however, the linear relation between the investigated values remained rather strong.

**Figure 4:**
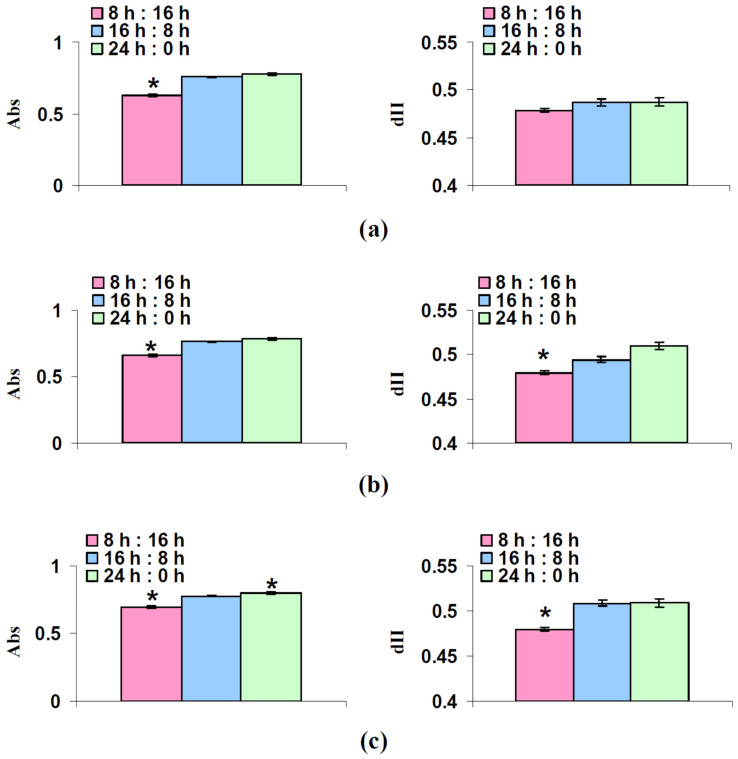
Fraction of the actinic light absorbed by the leaf (Abs) and fraction of the absorbed light distributed to photosystem II (dII) after 18 days (**a**), 25 days (**b**), and 32 days (**c**) of lettuce cultivation (*n* = 6). “8 h:16 h” is 8 h (light):16 h (dark), “16 h:8 h” is 16 h (light):8 h (dark), and “24 h:0 h” is 24 h (light):0 h (dark). *, difference from control plants cultivated under 16 h (light):8 h (dark) were significant (*p* < 0.05).

The long-term changes in LEF could be caused by changes in the fraction of the actinic light absorbed by the leaf (Abs) and the fraction of the absorbed light distributed to photosystem II (dII). The influence of photoperiod on these parameters was analyzed on the next stage of the current investigation. Figure 4 reports that cultivation under the 8 h photoperiod could decrease both Abs and dII (its decrease was not significant after 18 days of the cultivation); relative values of Abs and dII were 83–89% and 94–98%, respectively. Increasing the photoperiod slightly increased Abs and dII; however, the increase of Abs in plants after 32 days of the lettuce cultivation was significant. These results indicated that changes in dII and particularly Abs could be the mechanism of influence of the short photoperiod on the linear electron flow. 

The relative total contents of chlorophylls per leaf area in lettuce plants after 18, 25, and 32 days of cultivation under various photoperiods were measured using a chlorophyll meter. It should be noted that these measurements, which were based on values of light transmission at 620 nm (large light absorption by chlorophylls) and 940 nm (weak light absorption by chlorophylls), provided a rough estimate of the relative chlorophyll content; however, this widely-used method was high-throughput and provided a larger quantity of repetitions.

**Figure 5:**
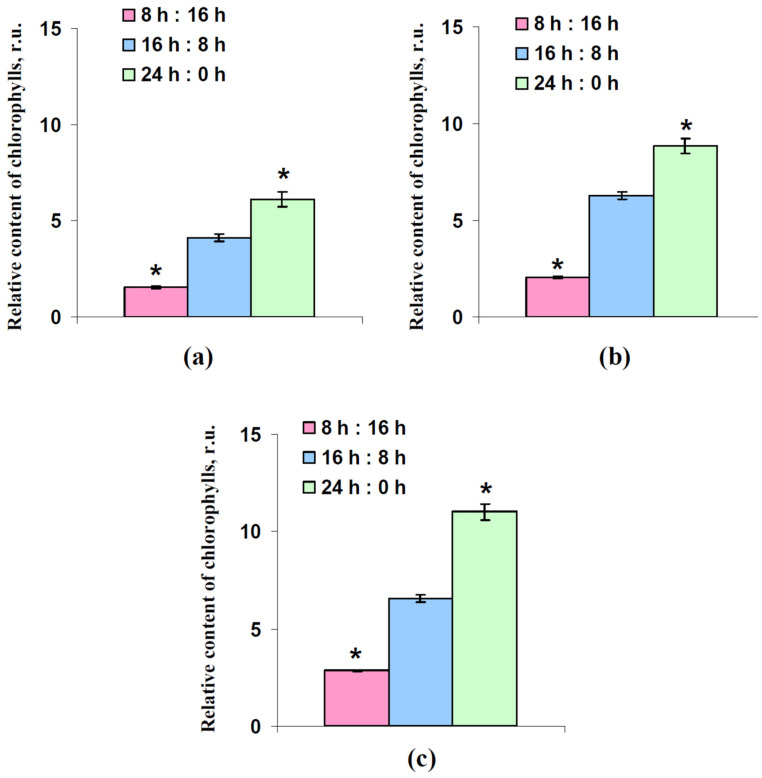
Relative total content of chlorophylls per leaf area after 18 days (**a**), 25 days (**b**), and 32 days (**c**) lettuce cultivation (*n* = 6). “8 h:16 h” is 8 h (light):16 h (dark), “16 h:8 h” is 16 h (light):8 h (dark), and “24 h:0 h” is 24 h (light):0 h (dark). *, difference from control plants cultivated under 16 h (light):8 h (dark) were significant (*p* < 0.05).

Figure 5 reports that the relative content of chlorophylls was strongly related to the photoperiod; all changes were significant in comparison to the control contents of chlorophylls in plants cultivated under the 16 h (light):8 h (dark) time regime of illumination. Compared to the control values, the chlorophyll contents were 33–43% and 141–167% under the 8 h and 24 h photoperiods, respectively. This result was in a good accordance with the literature, which showed an increasing chlorophyll content with increasing photoperiod [28,30].

**Figure 6:**
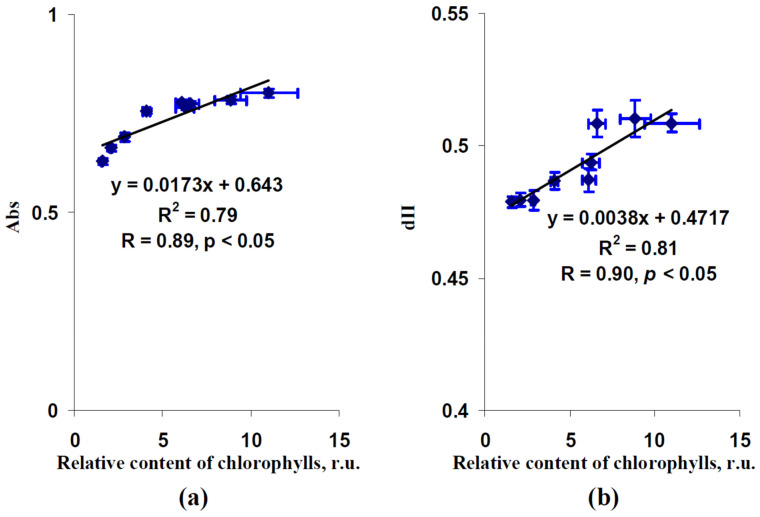
Dependences of the fraction of the actinic light absorbed by the leaves (Abs) (**a**) and fraction of the absorbed light distributed to photosystem II (dII) (**b**) on the relative content of chlorophylls in lettuce leaves. Average values from Figure 4 and Figure 5 were used. R^2^ and R are the determination and correlation coefficients.

Figure 6 shows scatter plots between the relative chlorophyll content and Abs and between this content and dII. Both scatter plots could be well described by linear regression equations (the determination coefficients were about 0.8); the investigated values were strongly and significantly correlated. These results showed that changes in the relative chlorophyll content can explain the influence of photoperiod on the fraction of the actinic light absorbed by the leaf and the fraction of the absorbed light distributed to photosystem II.

**Figure 7:**
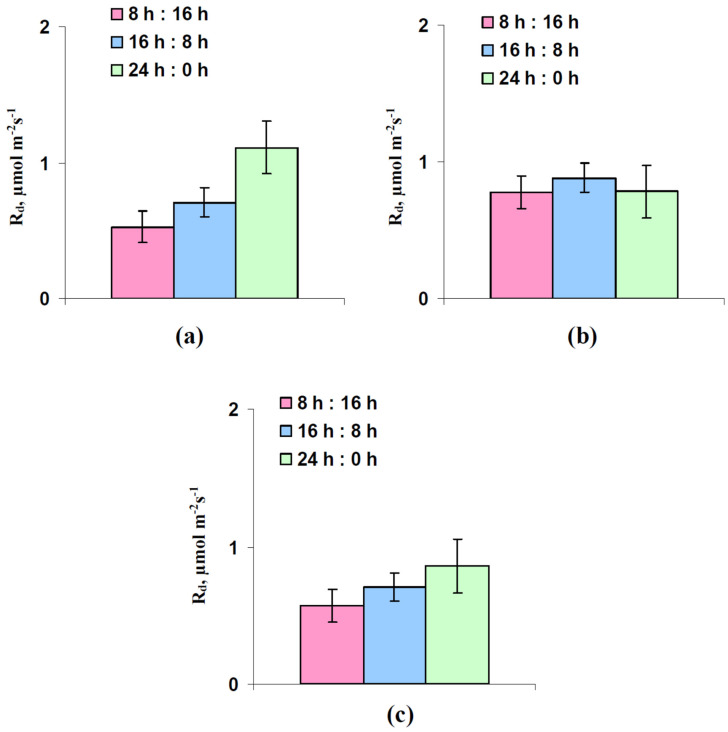
Influence of photoperiod on rate of the dark respiration rate (R_d_) after 18 days (**a**), 25 days (**b**), and 32 days (**c**) lettuce cultivation (*n* = 6). “8 h:16 h” is 8 h (light):16 h (dark), “16 h:8 h” is 16 h (light):8 h (dark), and “24 h:0 h” is 24 h (light):0 h (dark). Significant differences from control plants cultivated under 16 h (light):8 h (dark) were absent.

We previously showed that differences of the dark respiration rate (R_d_) in lettuce plants cultivated under light with different spectra were the basis of differences of DW in these plants. It is possible that the dark respiration rate was also the target of influence of the photoperiod; however, an experimental analysis (Figure 7) did not show significant differences of R_d_ in the lettuce plants cultivated under different photoperiods. There were only tendencies to increasing R_d_ with the increasing photoperiod in lettuce plants after 18 and 32 days of cultivation.

Our results suggest that lower DW in plants grown under the 8 h photoperiod could be explained by decreasing the chlorophyll contents, which suppressed photosynthetic activity through lowering the PAR absorption by leaves and decreasing the fraction of the absorbed light distributed to photosystem II. However, the photosynthetic CO_2_ assimilation rate and linear electron flow were not increased in plants cultivated under the 24 h photoperiod. This shows that the stimulation of production under these conditions had another cause. As a result, the next question was whether increasing the photoperiod stimulated an increase in DW without changes in photosynthesis and respiration?

### 2.3. Production Modeling

**Figure 8:**
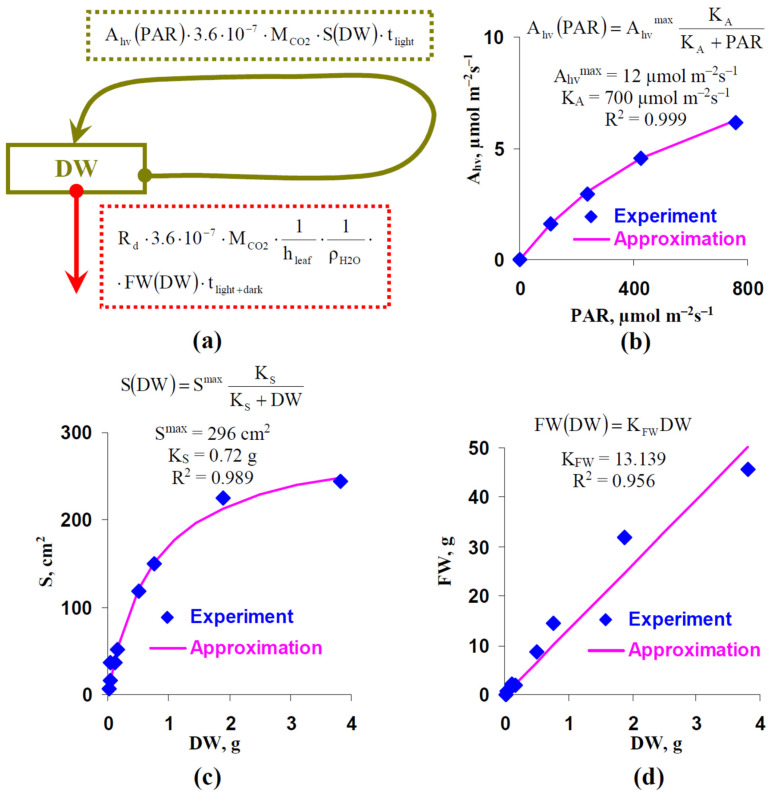
(**a**) Scheme of the simple model of plant production. DW is dry weight of shoot, Ahv(PAR) is the photosynthetic CO_2_ assimilation rate under this actinic light intensity (PAR), M_CO2_ is the molar mass of CO_2_, S(DW) is the green area per plant at this DW which shows the total area of illuminated leaves (or their parts) in the plant, t_light_ is the duration of light period at cultivation, t_light+dark_ is the total duration of light and dark periods at cultivation (24 h), 3.6 · 10^−7^ is correction coefficient for transition of units of A_hv_(PAR) from µmol m^−2^s^−1^ to mol cm^−2^h^−1^, R_d_ is the average dark respiration rate which was calculated on the basis of all respiration rates in our work (0.769 µmol m^−2^s^−1^), h_leaf_ is thickness of lettuce leaf (0.028 cm in accordance with [36]), ρ_H2O_ is the density of water, and FW(DW) is the total biomass of the plant (FW) at this DW. (**b**) The average light dependence of A_hv_ and its approximation. This dependence was calculated on the basis of all light dependences of A_hv_ measured in the current work. The standard equation from chemical kinetic was used for the approximation of A_hv_(PAR). A_hv_^max^ is the maximum rate of the photosynthetic CO_2_ assimilation, K_A_ is the light intensity at A_hv_(PAR) = 0.5 · A_hv_^max^, R^2^ is the determination coefficient for the approximation. (**c**) The average dependence of the green area per plant (S) on DW and its approximation. This dependence was calculated on basis of Figure 1 and Table 1 in the current work (all investigated variants). The standard equation from chemical kinetics was used for the approximation of S(DW). S^max^ is the maximum S, K_S_ is the DW at S(DW) = 0.5 · S^max^, R^2^ is the determination coefficient for the approximation. (**d**) Average dependence of FW on DW and its approximation. This dependence was calculated on the basis of Table 1 in the current work (all investigated variant). The standard linear equation was used for the approximation of S(DW). K_FW_ is the coefficient of the linear regression, R^2^ is the determination coefficient for the approximation.

A mathematical model of DW increase in lettuce was developed to analyze the effect of the photoperiod on plant production. Figure 8a shows the general scheme of the model, which is based on the difference between the production of DW (in the model we focused on the shoot dry weight) through photosynthetic processes and its utilization through respiration. Equation (1) was used for this model description:

(1) $$\begin{array}{l} \frac{\mathrm{dDW}}{\mathrm{dt}}=A_{\mathrm{hv}}\left(\mathrm{PAR}\right)\cdot 3.6\cdot 10^{-7}\cdot M_{\mathrm{CO}2}\cdot S\left(\mathrm{DW}\right)\cdot t_{\mathrm{light}}-\\ -R_{d}\cdot 3.6\cdot 10^{-7}\cdot M_{\mathrm{CO}2}\cdot \frac{1}{h_{\mathrm{leaf}}}\cdot \frac{1}{\rho_{H2O}}\cdot \mathrm{FW}\left(\mathrm{DW}\right)\cdot t_{\mathrm{light}+\mathrm{dark}} \end{array}$$

where A_hv_(PAR) was the dependence of the photosynthetic CO_2_ assimilation rate on the light intensity, 3.6 · 10^−7^ was the coefficient for transformation of units from “µmol m^−2^s^−1^” to “mol cm^−2^h^−1^”, M_CO2_ was the molar mass of CO_2_ (44 g mol^−1^), S(DW) was dependence of the total leaf area illuminated by the direct light (this value corresponds to the average green area per leaf measured on basis of photo and showed in Figure 1) on DW, t_light_ was the photoperiod (h), t_light+dark_ equaling 24 h was the total duration of the light-dark cycle, h_leaf_ was the leaf thickness which equaled 0.028 cm for lettuce plants [36], $\rho_{H2O}^{}$ was the water density (about 1 g cm^−3^), FW(DW) was dependence of biomass (fresh weight, FW) on DW, and R_d_ was the average dark respiration rate which was assumed as the constant. It should be noted that the final CO_2_ assimilation was calculated per area, and the final respiration was calculated per FW.

We approximated the experimental dependence of A_hv_ on PAR, which were averaged on the basis of all experimental variants (8, 16, and 24 h photoperiods) (Figure 8b) by using the standard equation from chemical kinetic (Equation (2)):

(2) $$A_{\mathrm{hv}}\left(\mathrm{PAR}\right)=A_{\mathrm{hv}}{}^{\max}\frac{\mathrm{PAR}}{\mathrm{PAR}+K_{A}}$$

where A_hv_^max^ was the maximum photosynthetic CO_2_ assimilation rate, K_A_ was the light intensity corresponding to A_hv_(PAR) = 0.5 · A_hv_^max^. Even if many alternative descriptions of Ahv(PAR) are available, particularly those based on the Farquhar, von Caemmerer, and Berry models [35,37] or on the rectangular hyperbolic models [38], we used the simplest description describing the experimental light dependence (R^2^ = 0.999), since it was fitting enough for our analysis.

The standard equation from chemical kinetic was also used for the description of S(DW) (Equation (3)); this description was based on the dependence of the average green area per plants on DW (Figure 8c) using results from Figure 1 and Table 1:

(3) $$S\left(\mathrm{DW}\right)=S^{\max}\frac{\mathrm{DW}}{\mathrm{DW}+K_{S}}$$

where S^max^ was the maximum S, K_S_ was the DW at S(DW) = 0.5 · S^max^. Equation (3) sufficiently described the experimental dependence (R^2^ = 0.989).

Finally, FW(DW) was described on the basis of a simple linear Equation (4):

(4) $$\mathrm{FW}\left(\mathrm{DW}\right)=K_{\mathrm{FW}}\mathrm{DW}$$

where K_FW_ was the coefficient of proportionality between FW and DW. Equation (4) well described the experimental dependence (R^2^ = 0.956).

**Figure 9:**
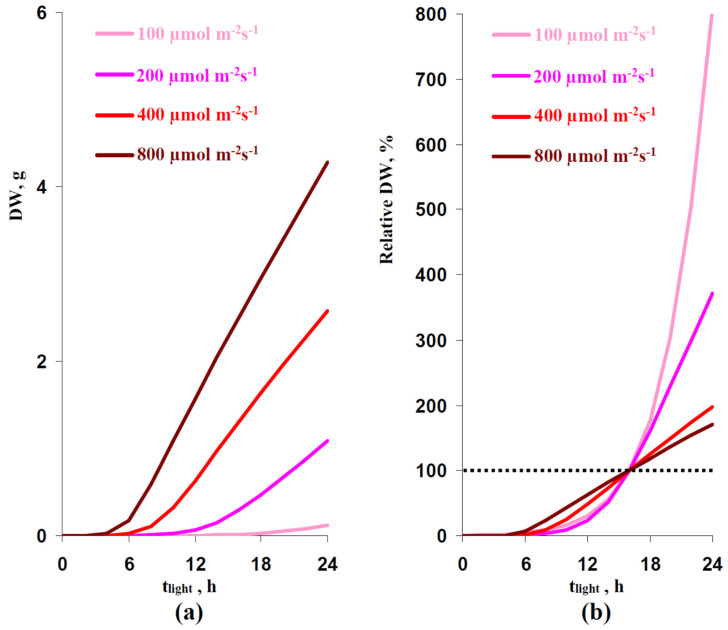
Dependences of the absolute (**a**) and relative (**b**) DW on photoperiod (t_light_) simulated by models under various intensities of the illumination. Relative DW was calculated as the percentage from the dry weight in the variant with 16 h (light):8 h (dark) illumination regime (the control variant). Simulated DW were estimated after 32 days of cultivation; initial DW (zero day) was assumed as the 0.01 g.

The model was numerically analyzed using the forward Euler method. It was assumed that the initial DW was 0.01 g. First, DWs after 32 days of lettuce cultivation under different photoperiods and different illumination intensities were analyzed (Figure 9).

A longer photoperiod strongly increased the simulated absolute (Figure 9a) and relative (Figure 9b) DW; dependences of DW on t_light_ were non-linear and this was in a good accordance with our experimental results (Table 1). Increasing the illumination intensity decreased the non-linearity of simulated dependences: this dependence was strongly non-linear under the 100 µmol m^−2^s^−1^ PAR and was moderately non-linear under the 800 µmol m^−2^s^−1^ PAR (Figure 9b).

The work [30] showed that increasing the lettuce production could be observed under the combination of the increased photoperiod and decreased light intensity during the lettuce cultivation; the total light integral for day (sum of illumination for one day) was not changed in this experiment. We analyzed the developed model to check these results. Table 2 reports that changes in the photoperiod could induce changes in DW at the constant total light integral. Increasing this integral decreased the relative value of the effect; however, it was expressive under all investigated light intensities.

Table S1 shows stationary DW under these light regimes. The influence of photoperiods on the stationary DW was observed without changes in the total light integral for day. The relative effect was weakly dependent on the value of this integral.

The results of the simulation shows that the positive and negative changes in DW under increased and decreased photoperiods, respectively, could appear without changes in photosynthetic processes. Moreover, the positive influence of increasing the photoperiod could be observed under the decreased photosynthetic CO_2_ assimilation (under the decreased PAR).

## 3. Discussion

The parameters of illumination are key factors of improving the photosynthesis and production of agricultural plants [2,3,18]; it is especially important for plants which are mainly cultivated under the artificial illumination including lettuce. We previously showed [22] that using blue, red, and white LEDs with high intensity of the red light is effective for the lettuce cultivation (cultivar “Azart”). This result was shown for plants cultivated under the 16 h (day):8 h (dark) time regime of illumination. It can be expected that increasing the photoperiod should additionally increase the lettuce production; however, the literature results seem to be contradictory. Work [29] shows that the increased photoperiod does not significantly change the lettuce production, and can even, decrease the linear electron flow; in contrast, some studies [28,30] reported a positive influence of the increased photoperiod on the photosynthetic parameters and production of lettuce plants. In this work we investigated the effect of photoperiod on the photosynthetic parameters, respiration, content of chlorophylls, biomass, and dry weight.

There are several important results shown in this work. First, the photoperiod strongly influences the lettuce production (the biomass and dry weight): the 24 h photoperiod is optimal for the lettuce production, and the 8 h photoperiod contributes extremely low biomass and dry weight of the plant shoots. This result is in a good accordance with several previous works [28,30,31] that investigated the lettuce cultivation and showed the positive influence of the photoperiod in production.

It is possible that increased photosynthesis (the stimulation of photosynthetic processes with increasing photoperiod is shown in several works [28,30,31]) can be the reason for the changes in production. Our analysis shows that decreasing photosynthesis under the decreased photoperiod can be a mechanism of suppression of production (Table 1, Figure 2); in contrast, increasing DW under the increased photoperiod is observed without significant changes in photosynthetic parameters (even, weak decreasing A_hv_ and LEF can be observed). Previously, we showed [22] that decreasing the dark respiration rate can be a mechanism of stimulation of the lettuce production under the use of illumination with the intensive red light. However, the influence of photoperiod on the dark respiration rate is also absent in the current work (Figure 7). Thus, there are at least two mechanisms of change in the lettuce production under changes in photoperiod: (i) based on decreasing photosynthesis (the production suppression under the 8 h photoperiod), and (ii) related to other mechanisms (the production stimulation under the 24 h photoperiod; it is possible that these mechanisms can additionally participate in the production suppression under the 8 h photoperiod).

**Figure 10:**
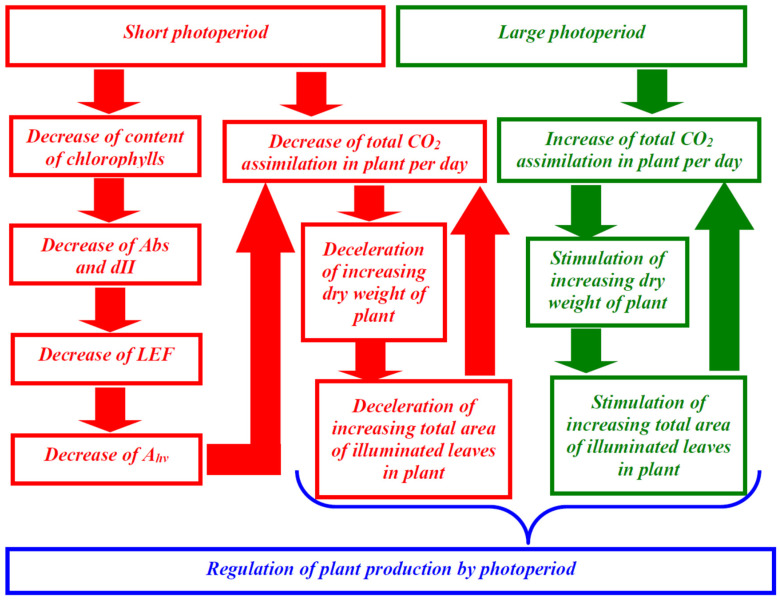
Potential ways of photoperiod influence on production in lettuce. The long photoperiod increases the total CO_2_ assimilation in the plant and thereby stimulates increased dry weight and the total area of illuminated leaves in the plant. This increasing additionally stimulates the total CO_2_ assimilation forming a positive feedback loop. In contrast, the short photoperiod decreases the total CO_2_ assimilation in the plant because the duration of photosynthetic activity is decreased, and duration of the dark respiration is not changed. This effect decelerates the increase in the dry weight and the total area of illuminated leaves in the plant. Additionally, this short photoperiod decreases the content of chlorophylls; this effect decreases Abs and dII and, thereby, suppresses the linear electron flow and CO_2_ assimilation.

Second, the linear dependence of A_hv_ on LEF (Figure 3) shows that the LEF suppression can be the potential mechanism of decrease of the photosynthetic CO_2_ assimilation. It should be noted that the linear dependences between these values can be observed [34,35] under widely ranging light intensity and medium CO_2_ concentration (350–400 ppm) in different plant species; these results additionally support the notion that changes in LEF can cause changes in A_hv_ (at least, in investigated conditions). In turn, LEF suppression can result from the decreasing of the fraction of the actinic light absorbed by the leaf and fraction of the absorbed light distributed to photosystem II (Figure 4), which are caused by lowering content of chlorophylls (Figure 5 and Figure 6). This potential mechanism (Figure 10) is in a good accordance with the decreasing content of chlorophylls in lettuce plants under the short photoperiod, which is shown in a number of works [28,30,39]; however, it should be noted that changes in the chlorophyll content are not shown in several works [29,40]. Increasing the chlorophyll content under the increased photoperiod can be caused by the direct light influence on the reduction of protochlorophyllide to chlorophyllide [41,42]; this hypothesis is in accordance with the negative influence of the increased photoperiod on the chlorophyll content under the constant total light integral for one day [30].

Third, analysis of the developed simple model of the lettuce production, which is based on the balance between the photosynthetic synthesis of biomass and its respiratory utilization, shows that changes in photoperiod can increase or decrease DW without additional changes in photosynthesis and respiration. The main assumptions of the model are (i) proportionality of the total utilization of organic compounds through respiration to the biomass of the plant (we consider the plant shoot only for simplification; however, using total biomass does not qualitatively change the model), (ii) the proportionality of the total photosynthetic production to the total area of leaves illuminated by the direct light (i.e., the measured average green area per plant) and to photoperiod, and (iii) increasing this total leaf area with increasing DW (we describe this increasing on basis of approximation of the experimental dependence of the average green area per plant on DW). It should be noted that the second assumption is simplification because the classical “sun–shade model” [43,44] describes the illumination of “sun” parts of canopy by direct light and illumination of “shade” parts by diffuse light; we assume that the diffuse light is low and can be eliminated from the model.

The combination of these assumptions provides fast increasing DW at even small changes in photoperiod, increasing the difference between the synthesis and utilization of biomass; however, saturation in dependence of the total area of illuminated leaves on DW prevents an infinite acceleration of biomass increase and limits production (Figure 10). This mechanism of photoperiod-caused changes in production is in accordance with the hypothesis about stimulation production through an expansion of the total leaf area with increasing photoperiod [42]. Potential mechanisms of this expansion include the simple increase of leaf size [45,46], possibly caused by the DW increase [42], the stimulation of division and expansion of cells [46,47,48], changes in in leaf anatomy [47,48], and others. It should be noted that the specific mechanism of increasing the leaf area is not crucial for the model: increasing the total area of directly illuminated leaves with increasing DW is sufficient for simulation. However, the results of simulation can depend on the shape of the dependence of the area of directly illuminated leaves on the total leaf area, which can be related to parameters of the plant canopy in accordance with the sun–shade model [43,44,49]. These parameters can regulate the photoperiod influence on DW; the effect can explain significant variety of this influence in different plant species [42].

Fourth, the analysis of the developed model shows that negative and positive effects of the photoperiod can be observed at the constant total light integral, showing the sum of light intensities for one day (Table 2). This result is in a good accordance (i) with our experimental results showing non-proportional changes in DW and this integral; and (ii) with literature data showing the increase of photoperiod without changes in the total light integral increases biomass, DW, leaf size, and other parameters related to plant growth and production [30,50,51]. This means that increasing the light intensity cannot be sufficient for the compensation of the photoperiod; in contrast, the increased photoperiod can effective compensate decreasing the light intensity. The photoperiod influence can be potentially compensated by long-term time intervals; however, analysis of the stationary DW simulated by the model (Table S1) also shows photoperiod-dependent changes in DW under the constant light integral for day. On the other hand, the total light integral certainly affects the productivity; this point is supported by the model analysis (Table 2 and Table S1) showing the increase of DW with increasing light intensity and without changes in the photoperiod. This effect of the total light integral is in good accordance with the literature data (see, e.g., [28]).

As a whole, the current investigation shows that changes in photoperiod can strongly influence the lettuce production (primarily, increasing dry weight). This influence is caused by changes in the chlorophyll content (under the short and moderate photoperiods) and by changes in area of illuminated leaves (under different photoperiods). The result can be important for lettuce cultivation under artificial illumination because it shows that a 24 h photoperiod is optimal for its production. The simple model of the lettuce production (the dry weight increase), which was developed and was used for analysis, is an additional result of the current work.

## 4. Materials and Methods

### 4.1. Plant Materials, Light Conditions, and General Schema of the Experiment

The green leaf lettuce (*Lactuca sativa* L.) cultivar “Azart” was cultivated in the vegetation room in accordance with our previous work [22]. Seeds were germinated for 3 days without illumination in pots containing a cube of mineral wool (1 plant per pot); 15 pots were placed on each pallet. Pallets with lettuce plants were then placed in the LED system. The average air temperature was about 23 °C; temperature deviation was within 2 °C. Day and night temperatures did not differ. The humidity was about 50%. Lettuce plants in all experimental variants were simultaneously cultivated to the maximal standardization of experimental conditions.

The Medium Flora Series^®^ (Terra Aquatica, Fleurance, France) was used for cultivation. The plants were irrigated by this medium every day. In the specific pot, the irrigation was terminated after the termination of absorption of the medium into mineral wool (i.e., the maximal medium content was provided).

**Figure 11:**
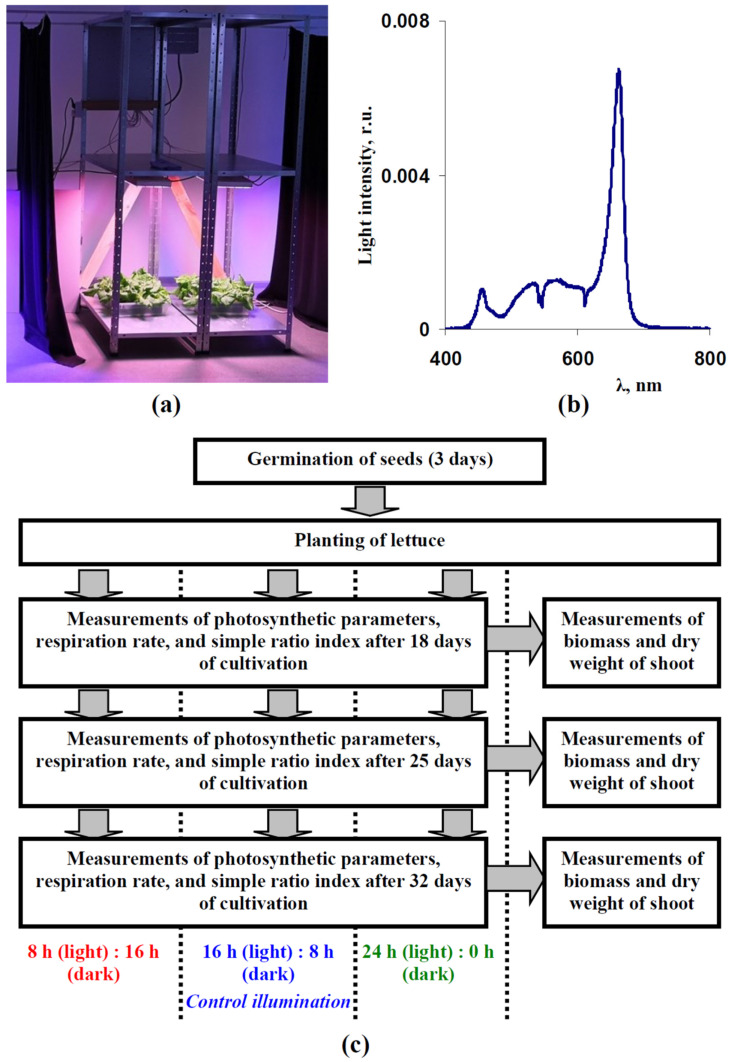
(**a**) Equipment used for lettuce cultivation under controlled light spectrum, intensity and photoperiod. (**b**) The illumination spectrum which was used for the lettuce cultivation. The spectrum was normalized on the total sum of intensities within 400–800 nm. The total light intensity was about 180 µmol m^−2^s^−1^. (**c**) General scheme of the lettuce cultivation and measurements of photosynthetic parameters, dark respiration rate, relative chlorophyll content, biomass, and dry weight (see Section 4 for details). Measurements of green area per plant, which were periodically performed after 10 days of the cultivation, were not shown in the scheme.

The previously-developed plant illumination system (see [22] for details) was used as a light source (Figure 11a). This system included 4000 K white LEDs, blue LEDs with maximums at 440–460 nm, red LEDs with maximums at 630–660 nm, and far-red LEDs with maximums at 730–740 nm (VANQ technology Co., Ltd., Shenzhen, China) with regulated intensities. In accordance with Yudina et al. [22], we used the combination of blue, white, and red LEDs with high intensity of the red light for the lettuce cultivation; the used spectrum is shown in Figure 11b. A FLAME-S-VIS-NIR spectrometer (Ocean Optics, Dunedin, FL, USA) was used to control this spectrum. The light intensity was 180 µmol m^−2^s^−1^; the Thorlabs PM100D optical power meter (Thorlabs Inc., Newton, MA, USA) with an S120VC sensor (200–1100 nm) was used to control the light intensity. This light intensity was used on the basis of our previous works [22,29] and results by Iqbal et al. [28]; the last work showed that light dependences of biomass and DW of leaves in lettuce plants were saturated under light intensity equaling to 200 µmol m^−2^s^−1^(14 and 17 h photoperiods, illumination by LEDs).

Three variants of photoperiods were used (Figure 11c): 8 h (light):16 h (dark), 16 h (light):8 h (dark), and 24 h (light):0 h (dark). Photosynthetic parameters, respiration rate, relative chlorophyll content, biomass, and dry weight were measured after 18, 25, and 32 days of the lettuce cultivation. The average green areas per plant were measured at the same time points and, additionally, in 11, 16, and 20 days after transplant.

### 4.2. Photosynthetic Parameters and Dark Respiration Rate

The parameters for photosynthesis and respiration were measured using the standard system (Heinz Walz GmbH, Effeltrich, Germany): the gas analyzer GFS-3000, PAM-fluorometer Dual-PAM-100, and common measuring head Dual-PAM gas-exchange Cuvette 3010-Dual. This system provided a 360 ppm CO_2_ concentration, the H_2_O concentration was 20,000 ppm, and the temperature was 23 °C. Weak pulses of blue light (460 nm) were used as the measuring light; pulses of red light (630 nm, 300 ms, 10,000 µmol m^−2^s^−1^) were used as the saturation light. Blue light with different intensities was used as the actinic light.

Parameters of photosynthetic light reactions were measured by using Dual-PAM-100. Measurements were initiated after 15 min dark adaptation; saturation pulses were generated every 20 s. The effective quantum yields of PSI (Φ_PSI_) and PSII ((Φ_PSII_)) were automatically calculated by the Dual-PAM-100 software in accordance with widely used equations [20,52,53,54] on the basis of the chlorophyll fluorescence and light absorption parameters at 830 and 870 nm.

Equation (5) was used to calculate the LEF in an accordance with Yudina et al. [22]:

(5) $$\mathrm{LEF}=\mathrm{Abs}\cdot \mathrm{PAR}\cdot \mathrm{dII}\cdot \Phi_{\mathrm{PSII}}$$

where PAR was the intensity of the actinic light, Abs was the fraction of the actinic light absorbed by the leaves, dII was the fraction of the absorbed light distributed to photosystem II, and (1-dII) was the fraction of the absorbed light distributed to photosystem I. Abs was calculated as SR−1SR where SR was the ratio of the leaf reflectance for the near infrared light (about 760–790 nm [55]) to this reflectance for the photosynthetically active red light (about 660–680 nm [55]). SR was measured with using the handheld PolyPen RP 410 UVIS systems (Photon Systems Instruments, Drásov, Czech) which could measure the leaf reflectance, absorbance, and transmittance. It should be noted that the calculation of Abs on the basis of reflectance at the near infrared and red spectral range is a widely used approach for the simple estimation of this absorption [56]. Calculated Abs could be used for absorption of blue light because light absorbance at the photosynthetically active blue light (about 460 nm for Dual-PAM-100), and this absorbance at the photosynthetically red light (about 660–680 nm) had similar values and were strongly related in a linear manner (Figure S2).

The dII was calculated as ΦPSIΦPSI+ΦPSII, where both Φ_PSI_ and Φ_PSII_ were measured under the low intensity of the actinic light [22,56,57]. It should be noted that although Equation (5) was the conventional equation which was widely used for the description of LEF (see, e.g., [58,59,60]), some works provided ways of improving this equation to a more accurate estimation of additional electron flows through photosystem II (mainly, pseudocyclic electron flow) [61,62]. However, using this improved equation required the experimental estimation of additional parameters that restricted the application of this method. As a result, we used conventional Equation (5) in our investigation.

The CO_2_ assimilation (A) was measured on the basis of GFS-3000. A_hv_ was calculated as the difference between A under the actinic light and A under dark conditions (after the termination of illumination). The dark respiration rate (R_d_) was calculated as -A under dark conditions.

The dependences of A_hv_ and LEF on the actinic light intensity were analyzed at 0, 108, 239, 425, and 758 µmol m^−2^s^−1^ intensities of the actinic light; the duration of illumination by each intensity was 200 s.

### 4.3. Relative Chlorophyll Content in Leaves

We used a standard chlorophyll meter CL-01 (Hansatech Instruments, Norfolk, UK) for the high-throughput investigation of relative total chlorophyll content per leaf area in lettuce. Measurements of the relative chlorophyll content were based on measurements of light transmission at 620 nm (large light absorption by chlorophylls) and 940 nm (weak light absorption by chlorophylls); it is known that relative values measured by CL-01 are strongly related in a linear manner to the content of the concentration of chlorophylls per leaf area estimated by biochemical methods [63].

### 4.4. Biometric Determinations

The average green area per plant was calculated on the basis of photos of the pallets with the lettuce plants (vertical position, same distance between pallet and camera, and black background were used). ImageJ 1.46r was used for calculation of these areas in accordance with Yudina et al. [22]. This average green area per plant showed the average area of illuminated parts of leaves in lettuce plants (for vertical illumination used in our work). In contrast, the relation of this green area to total leaf area in plant should be non-linearly changed with lettuce growth.

The biomass and dry weight of separate plant shoots were measured to estimate the lettuce production. The dry weight was measured after 6 h of drying at 100 °C [22].

### 4.5. Statistical Analyses

Different lettuce plants were used in different experiments. Mean values, standard errors, determination and correlation coefficients were calculated. The significance of changes were estimated using a Student’s *t*-test.

## Figures and Tables

**Table 1:** Biomass and dry weight (DW) of shoots in plants after 18, 25, and 32 days of cultivation (*n* = 6). “8 h:16 h” is 8 h (light):16 h (dark), “16 h:8 h” is 16 h (light):8 h (dark), and “24 h:0 h” is 24 h (light):0 h (dark).

Duration of Cultivation	Parameter	8 h:16 h	16 h:8 h	24 h:0 h
18 days	Biomass, g	0.092 ± 0.01 *	0.725 ± 0.040	1.898 ± 0.480
	DW, g	0.010 ± 0.001 *	0.037 ± 0.009	0.160 ± 0.041 *
25 days	Biomass, g	0.609 ± 0.099 *	8.668 ± 0.764	14.457 ± 2.636
	DW, g	0.037 ± 0.008 *	0.503 ± 0.048	0.770 ± 0.072 *
32 days	Biomass, g	2.271 ± 0.302 *	31.773 ± 1.539	45.590 ± 6.594
	DW, g	0.119 ± 0.026 *	1.883 ± 0.113	3.822 ± 0.625 *

*, difference from control plants cultivated under 16 h (light):8 h (dark) was significant (*p* < 0.05).

**Table 2:** Dry weights (DW) of plants which were simulated by the model after 32 days of cultivation at various combinations of light intensity and duration of illumination during the day. Relative DW was calculated as a percentage from the control DW in plants cultivated under 16 h (light):8 h (dark) illumination regime.

Total Light Integral for Day, mol m^−2^ Day^−1^	Light Intensity, µmol m^−2^s^−1^	Duration of Illumination for Day, h	DW, g	Relative DW, %
	100	24	0.127	138
8.64	150	16	0.092	100
	300	8	0.039	42
	200	24	1.085	130
17.28	300	16	0.837	100
	600	8	0.344	41
	300	24	1.923	128
25.92	450	16	1.508	100
	900	8	0.697	46

## Data Availability

The data presented in this study are available upon request from the corresponding author.

## Supplementary Materials

The following supporting information can be downloaded at: https://www.mdpi.com/article/10.3390/plants12030442/s1, Figure S1: Scatter plot between average photosynthetic CO_2_ assimilation rate (A_hv_) and average linear electron flow (LEF) in lettuce plants under 8, 16, and 24 h photoperiods (*n* = 9) (a) and under 16 and 24 h photoperiods (*n* = 6) (b); Figure S2: Scatter plot of absorbance at 660–680 nm and absorbance at 460 nm in lettuce leaves; Table S1: Stationary dry weights (DW) of plants which were simulated by the model after cultivation at various combinations of light intensity and duration of illumination for day.
