# Supplementary material for: Effect of Duration of LED Lighting on Growth, Photosynthesis and Respiration in Lettuce
Source: Plants (Basel). 2023 Jan 18;12(3):442. doi: 10.3390/plants12030442 (PMC9921278; doi:10.3390/plants12030442)
Supplement: Supplementary file 1 [file plants-12-00442-s001.zip › Figure S1.pdf]

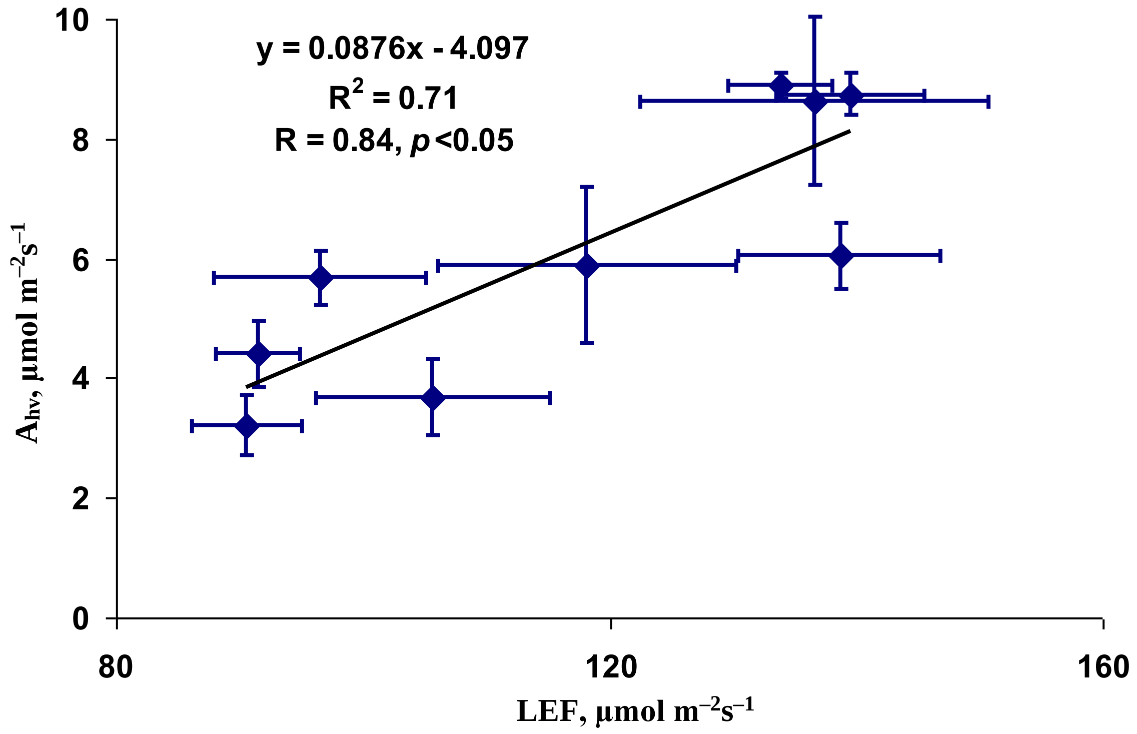

(a)

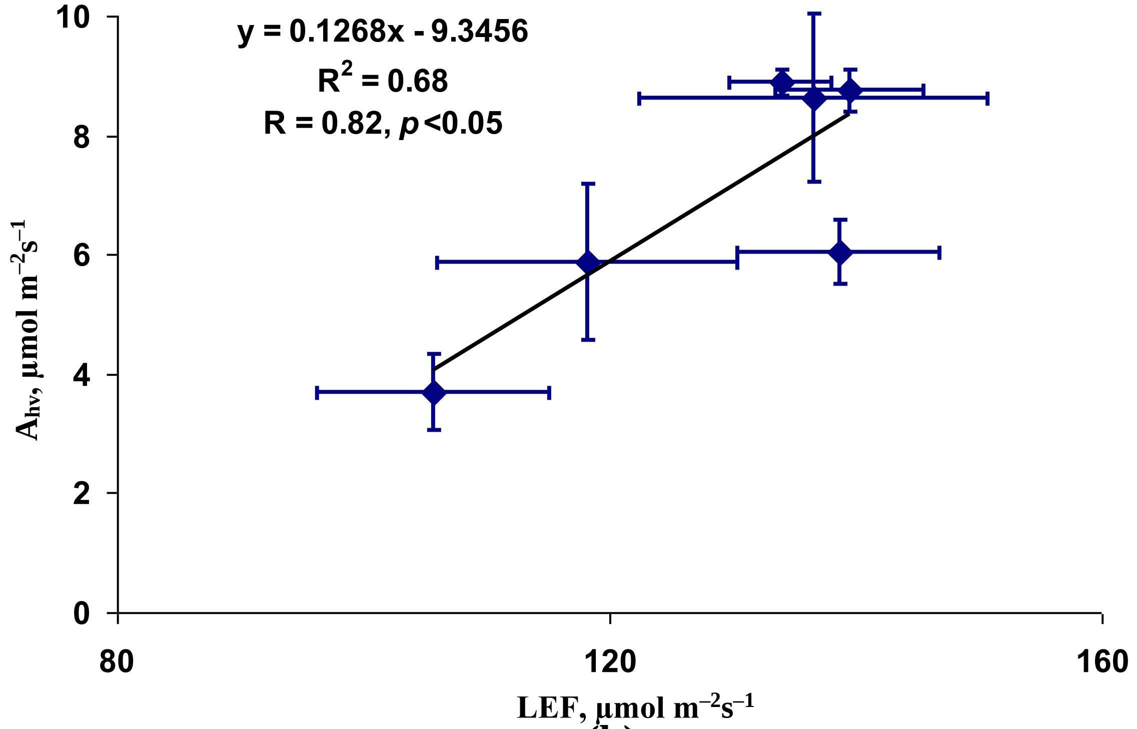

(b)

Figure S1. Scatter plot between average photosynthetic CO_2 assimilation rate (A_{hv}) and average linear electron flow (LEF) in lettuce plants under 8, 16, and 24 h photoperiods (n=9) **(a)** and under 16 and 24 h photoperiods (n=6) **(b)**. Only 758 µmol $m^{-2}s^{-1}$ light intensity was analyzed. R^2 and R are determination and correlation coefficients.
