# Supplementary material for: Effect of Duration of LED Lighting on Growth, Photosynthesis and Respiration in Lettuce
Source: Plants (Basel). 2023 Jan 18;12(3):442. doi: 10.3390/plants12030442 (PMC9921278; doi:10.3390/plants12030442)
Supplement: Supplementary file 1 [file plants-12-00442-s001.zip › Figure S2.pdf]

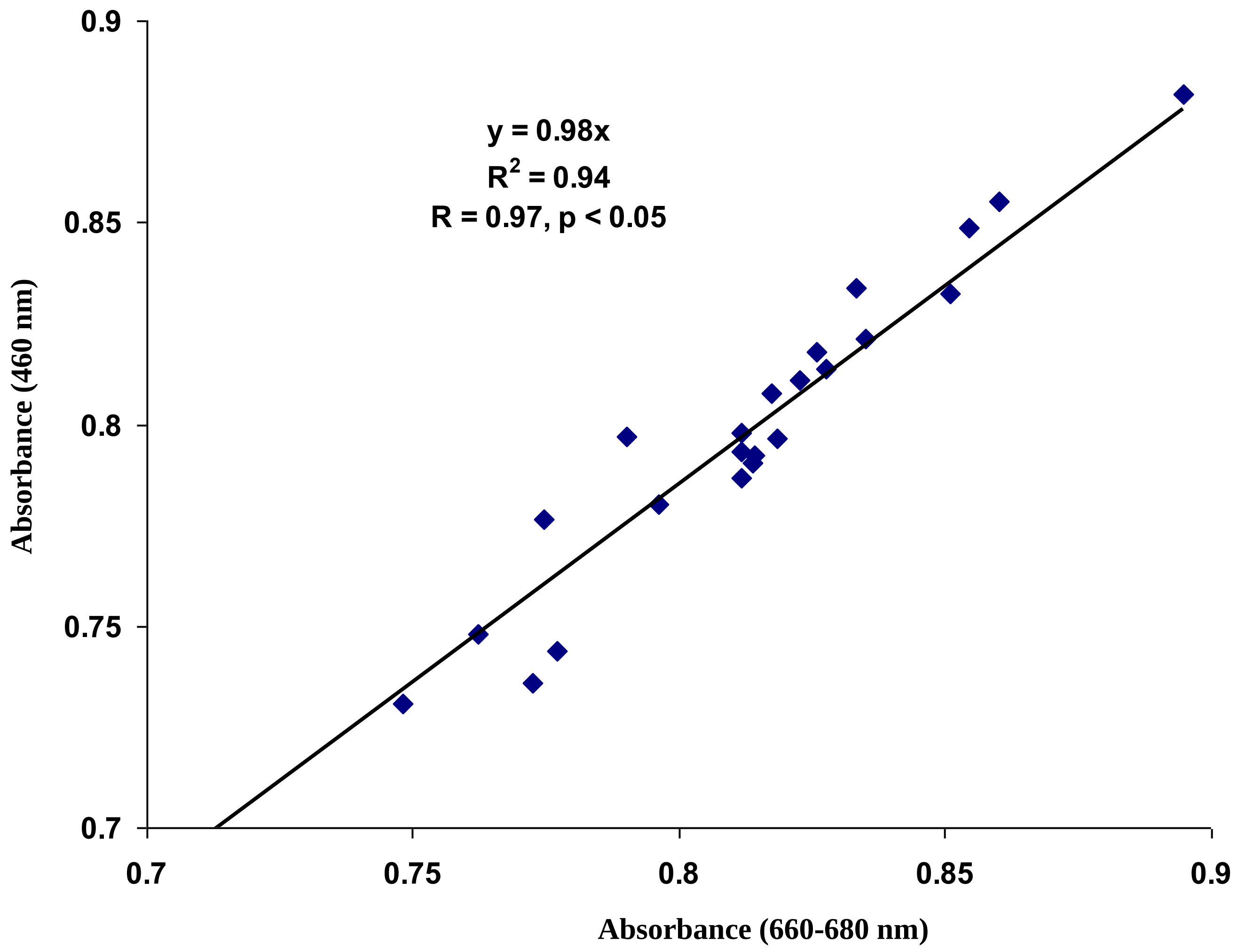

Figure S2. Scatter plot of absorbance at 660-680 nm and absorbance at 460 nm in lettuce leaves. Each point shows measurement in individual leaf with using PolyPen RP 410 UVIS. Lettuce plants cultivated under the 16 h photoperiod were investigated. R^2 and R are the determination and correlation coefficients.
