# Supplementary material for: Effect of Duration of LED Lighting on Growth, Photosynthesis and Respiration in Lettuce
Source: Plants (Basel). 2023 Jan 18;12(3):442. doi: 10.3390/plants12030442 (PMC9921278; doi:10.3390/plants12030442)
Supplement: Supplementary file 1 [file plants-12-00442-s001.zip › Table S1.pdf]

Table S1. Stationary dry weights (DW) of plants which were simulated by the model after cultivation at various combinations of light intensity and duration of illumination for day. Relative DW was calculated as percentage from the control DW in plants cultivated under 16 h (light) : 8 h (dark) illumination regime.

Total light integral for day, mol $m^{-2}day^{-1}$	Light intensity, μmol $m^{-2}s^{-1}$	Duration of illumination for day, h	DW, g	Relative DW, %
8.64	100	24	0.500	117
	150	16	0.427	100
	300	8	0.254	59
17.28	200	24	1.456	118
	300	16	1.238	100
	600	8	0.784	63
25.92	300	24	2.221	121
	450	16	1.836	100
	900	8	1.115	601

Thus, results of simulation shows that positive and negative changes in DW under increased
